# Arterial Stiffness Use for Early Monitoring of Cardiovascular Adverse Events due to Anthracycline Chemotherapy in Breast Cancer Patients. A Pilot Study

**DOI:** 10.5935/abc.20180168

**Published:** 2018-11

**Authors:** Cláudio Antônio de Souza, Ricardo Simões, Karina Braga Gomes Borges, Angélica Navarro de Oliveira, Juliana Barroso Zogeib, Bruno Alves, Marcus Vinicius Bolívar Malachias, Ana Paula Drummond-Lage, Bruno Almeida Rezende

**Affiliations:** 1 Faculdade de Ciências Médicas de Minas Gerais, Belo Horizonte, MG - Brazil; 2 Hospital Alberto Cavalcanti, Belo Horizonte, MG - Brazil; 3 Universidade Federal de Minas Gerais (UFMG), Belo Horizonte, MG - Brazil

**Keywords:** Breast Neoplasms, Vascular Stiffness, Stroke Volume/drug effects, Cardiotoxicity, Doxorubicin/adverse effects, Cyclophosphamide/adverse effects

## Abstract

**Background:**

Chemotherapy with doxorubicin and cyclophosphamide, although efficient for treating breast cancer, is associated with cardiovascular complications. Recent studies seek to identify methods that can early detect cardiological and vascular changes as a strategy to decrease the incidence of cardiovascular comorbidities.

**Objective:**

To evaluate the role of arterial stiffness measurement in the monitoring of doxorubicin and cyclophosphamide-induced cardiotoxicity in breast cancer patients.

**Methods:**

Prospective longitudinal study in 24 breast cancer patients undergoing treatment with doxorubicin and cyclophosphamide. Patients underwent an indirect evaluation of arterial stiffness through non-invasive measurement of hemodynamic parameters such as pulse wave velocity with the Mobil-O-Graph® 24H PWA device at three different times of the chemotherapy treatment (pre-chemotherapy, after the first and the fourth cycle). The left ventricular ejection fraction was also evaluated by Doppler echocardiography (pre-chemotherapy and after the fourth chemotherapy cycle). Data were considered significant when p ≤ 0.05.

**Results:**

Patients had a mean age of 52.33 ± 8.85 years and body mass index of 31 ± 5.87 kg/m^2^. There was no significant difference between the hemodynamic parameters evaluated by the oscillometric method or in the left ventricular ejection fraction in the different evaluated periods.

**Conclusion:**

Evaluations of arterial stiffness by oscillometry and measurement of left ventricular ejection fraction by Doppler echocardiography showed equivalence in the values found, suggesting that the evaluation method of arterial stiffness studied could be used as a marker for cardiovascular adverse events associated with doxorrubicin-based chemotherapy drugs.

## Introduction

Breast cancer is the most common cancer among women in Brazil and in the world, second only to non-melanoma skin cancer, accounting for approximately 25% of new cases each year.^1^ Advances in cancer therapy have resulted both in the improvement of quality of life and in the increase of cancer patients survival.^2^ However, in spite of the evolution in the pharmacological treatment of the different neoplasms, several studies have indicated a significant increase in the occurrence of cardiovascular adverse events, mainly myocardial dysfunction in patients undergoing chemotherapy with cardiotoxic drugs, such as the anthracycline group and, to a lesser extent, cyclophosphamide.^3^^-^^5^ Chemotherapy regimens using doxorubicin and cyclophosphamide are the most commonly used in the treatment of breast cancer in Brazil.^6^ The cardiotoxic potential of these drugs is already established, and it is evaluated mainly by Doppler echocardiography in studies showing an increase in the incidence of Heart Failure (HF) in patients who received these drugs.^7^

The early identification of the appearance of cardiovascular alterations in patients during chemotherapy with drugs considered cardiotoxic could help adjust cancer treatment, with the adoption of preventive, substitutive measures or their interruption, aiming at minimizing cardiovascular adverse events caused by these agents.^7^^,^^8^

Arterial Stiffness (AS) is characterized by the reduction of the arteries elastic properties due to intrinsic structural or functional changes.^9^ Aging is a normal evolutionary factor for vascular stiffening, and can be accelerated by several factors, such as diabetes and hypertension.^10^

Several studies have related the increase in AS with the progression of cardiovascular diseases.^3^^,^^5^^,^^11^ The early increase in AS can be estimated mainly through the evaluation of the Pulse Wave Velocity (PWV) obtained from indirect imaging or hemodynamic methods.^3^^,^^12^^,^^13^

Because cardiovascular changes are observed in some patients on doxorubicin, and AS measurement allows the detection of the onset and progression of cardiovascular disease, this study is warranted because it aims to estimate AS, based on PWV measurement, through oscillometric evaluation of the brachial artery, in patients with breast cancer in the initial phases of chemotherapy with doxorubicin combined with cyclophosphamide (AC regimen). In addition, it proposes to check if there is a correlation between AS and the values of Left Ventricular Ejection Fraction (LVEF), an altered condition in patients with cardiotoxicity due to chemotherapy.

## Methods

This is a prospective and longitudinal study with a convenience sample. Twenty-four women aged over 18 years with breast cancer and indication of at least four cycles (every 3 weeks) of adjuvant or neoadjuvant chemotherapy based on the AC regimen (at doses of 75 mg/m^2^ for doxorubicin and 600 mg/m^2^ for cyclophosphamide in each cycle, totaling 300 mg/m^2^ and 2,400 mg/m^2^ for doxorubicin and cyclophosphamide, respectively) were followed. The recruitment took place in an Oncology Outpatient Clinic of a High Complexity Unit in Public Oncology of the city of Belo Horizonte (state of Minas Gerais), from July 2016 to December 2017.

The following were excluded: pregnant and lactating women; patients with previous history of chemotherapy or radiotherapy; pre-chemotherapy assessment showing abnormal left ventricular systolic function (LVEF < 50%) evaluated by Doppler echocardiography; history of/or active heart disease; moderate to severe hepatic or renal dysfunction; brain-degenerative diseases requiring caregiver's action; and those in use of other chemotherapeutics other than the AC regimen in the treatment of breast cancer.

Randomization took place in an outpatient basis, under clinical evaluation by a cardiologist with experience in the area. Subsequently, patients underwent an echocardiographic study, according to the methodology proposed by Campos-Filho et al.,^14^ to evaluate cardiac parameters that could contraindicate participation in the study and also to monitor the cardiac function at different treatment times of chemotherapy, as suggested by current guidelines.^5^^,^^7^ Following these procedures, patients were referred for chemotherapy with doxorubicin and cyclophosphamide in the same hospital.

Brachial artery AS measurement was performed using the non-invasive device Mobil-O-Graph® 24h PWA (IEM, Germany) through oscillometric measurements on the upper limb. The device has a device for Blood Pressure (BP) measurement and provides measures of PWV, systolic and central diastolic pressure, and *augmentation index*, which are used as an estimate of AS. This device was validated for use in scientific research by the *European Society of Hypertension*.^13^ Measurements were made in the contralateral upper limb on the side affected by the tumor, seeking to exclude the influence of axillary dissection surgery and consequent lymphedema. After measuring the circumference of the limb and choosing the appropriate cuff, the device was positioned similarly to procedures defined by guidelines of cardiology societies.^7^ Mobil-O-Graph® 24h PWA is able to offer a number of useful results of the cardiovascular condition of the evaluated patient, because the BP and PWV measurements are correlated with the weight, height and age data previously provided by the HMS Client-Server data management software.

Follow-up chronology was implemented with measurements of hemodynamic parameters by Mobil-O-Graph® 24H PWA at three different times: (1) prior to chemotherapy, when measurements of the hemodynamic parameters through the oscillometric method were taken 15 minutes before the beginning of chemotherapy infusion; (2) post-1chemo, measured up to 30 minutes after intravenous (IV) infusion of the first cycle of the AC regimen; there was a variation of 45 to 90 minutes in the chemotherapy infusion; and (3) post-4chemo, measured up to 30 minutes after IV infusion of the fourth cycle of the AC regimen; the time interval from the start of chemotherapy to its completion was 80 to 90 days.

After 1 week of the fourth chemotherapeutic cycle, the patient underwent a new clinical-cardiological evaluation and an echocardiographic study for LVEF analysis and for comparison with the value before the first cycle.

The results of all the variables that the Mobil-O-Graph® 24H PWA instrument provided were tabulated and submitted to statistical treatment among the three measures of the patients studied.

The protocol of this study is in accordance with the Declaration of Helsinki, having been released by the Research Ethics Committee of the institution, and all the patients evaluated signed the Free Informed Consent Form (FICF).

### Statistical analysis

The variables underwent the Shapiro-Wilk normality test and were presented as mean ± Standard Deviation (SD), in case of normality, or as median (Interquartile Distance - DI - which is the difference between the third and the first quartiles). Categorical variables were expressed in frequency. The three measurements provided by the device were expressed as mean ± SD. In the comparison between the three moments (pre-chemotherapy, soon after the first cycle of chemotherapy, and after the fourth cycle), we adopted the analysis of variance for repeated measures, with the sphericity check, or Friedman test. The comparison of measurements between two moments was performed by the Wilcoxon test for paired samples, including post hoc analysis. The analysis was developed in the free software R, version 3.3.2, with a significance level of 5% being adopted.

## Results

The sample consisted of 24 women, mean age of 52.33 ± 8.85 years, and mean Body Mass Index (BMI) of 31 ± 5.87 kg/m^2^. Approximately 16.7% of the women were alcoholics, and 20.8% were smokers. More than half of them (58.3%) had hypertension, while 12.5% had type 2 diabetes mellitus (Table 1).

**Table 1:** Characteristics of the patients evaluated in the sample

Variables	n = 24
Age, years	52,33 ± 8,85
BMI, kg/m^2^	31 ± 5,87
Smoking	5 (20,8)
Alcoholism	4 (16,7)
Diabetes Mellitus	3 (12,5)
Hypertension	14 (58,3)

Results expressed as mean ± standard deviation or n (%). BMI: body mass index.

**Figure 1:**
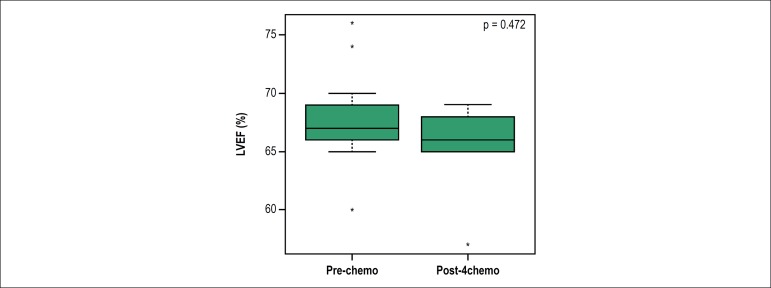
Left ventricular ejection fraction (LVEF) values measured by transthoracic Doppler echocardiography in breast cancer patients before (pre-chemo) and after the fourth cycle of chemotherapy (post-4chemo) in the chemotherapy regimen with doxorubicin combined with cyclophosphamide. p values refer to Wilcoxon test.

**Figure 2:**
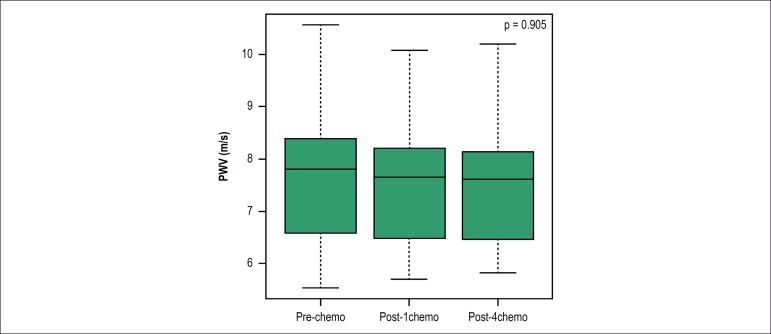
Box diagrams for pulse wave velocity (PWV) at the three times assessed: before, after the first cycle of chemotherapy, and after the fourth cycle (pre-chemo, post-1chemo, and post-4chemo). p value refers to analysis of single-factor variance.

LVEF mean values obtained through transthoracic Doppler echocardiogram before and after the fourth cycle of chemotherapy were 67.8% ± 3% and 66.0% ± 3%, respectively, and showed no significant difference between the two times (Figure 1). We also did not observe differences in hemodynamic variables among the three periods analyzed (pre-chemo, post-1chemo, and post-4chemo - all with p > 0.05), in relation to peripheral and central systolic and diastolic BP parameters, mean BP, pulse pressure, heart rate, pulse pressure augmentation, systolic volume, cardiac output, total vascular resistance, cardiac index, augmentation pressure, reflection coefficient and augmentation index (Table 2). PWV, a variable that correlated most with arterial stiffness, did not show a statistically significant difference among the three periods analyzed, with p = 0.507 (Figure 2).

**Table 2:** Longitudinal evaluation of heart parameters

Hemodynamic variables	Pre-chemo	Post-1chemo	Post-4chemo	p Value
Peripheral SBP, mmHg	125.7 ± 17	123.3 ± 18.2	123.7 ± 8.3	0.244^*^
Peripheral DBP, mmHg	79.9 ± 14	78.4 ± 10.2	80 ± 11.7	0.988^*^
Mean blood pressure, mmHg	100.3 ± 11.2	98.6 ± 11.4	100.3 ±10.1	0.879^†^
PP, mmHg	45.8 ± 12.4	42.5 ± 16.1	43 ±7.6	0.527^*^
Heart rate, bpm	76.4 ± 18.1	73.9 ± 16.8	78 ±15.7	0.055^*^
Central SBP, mmHg	117.1 ± 14	115.3± 13.3	116.2± 9.7	0.731^†^
Central DBP, mmHg	79.7± 10.7	79.5 ± 10.9	81.8 ± 10.7	0.815^†^
PP^N^ amplification	1.30 ± 0.11	1.25 ± 0.10	1.28 ± 0.10	0.428^†^
Stroke volume,mL/m^2^	67.4 ± 14.5	68.2 ± 13.5	64.4 ±11.8	0.144^†^
Cardiac output, L/minute	5.1 ± 0.6	4.9 ± 0.6	5 ± 0.5	0.521^†^
Total vascular resistance, mmHg/mL	1.2 ± 0.14	1.25 ± 0.16	1.24 ± 0.22	0.675^*^
Cardiac index, L/min/m^2^	2.8 ± 0.3	2.7 ± 0.5	2.7 ± 0.4	0.918^*^
Augmentation pressure, mmHg	8.8 ± 6.1	7.7 ± 5.1	7.7 ± 3.3	0.110^*^
Reflection coefficient, %	67.2 ± 7	69.8 ± 6.1	67.6 ± 6.2	0.136^†^
Augmentation index	26.6 ± 10.8	23.2 ± 11.6	24.4 ± 10.6	0.144^†^
PWV,m/s	7.61 ± 1.28	7.49 ± 1.20	7.45 ± 1.15	0.507^†^

Results expressed as mean ± standard deviation, or median ± difference between the third and first quartiles. For all measured variables, there were three missing data on the measurements after four cycles.

* Friedman test;

† analysis of variance for repeated measures. Pre-chemo: before chemotherapy; post-1chemo: after the first cycle of chemotherapy; post-4chemo: after the fourth cycle of chemotherapy; SBP: systolic blood pressure; DBP: diastolic blood pressure; PP: pulse pressure; PWV: pulse wave velocity.

## Discussion

Since the 1970s, chemotherapy with doxorubicin is known to be related to an increase in the prevalence of HF.^15^^-^^17^ To a lesser extent, but used in high doses, cyclophosphamide has also been shown to be toxic to the cardiovascular system.^18^ Currently, the main guidelines for chemotherapy of the most prevalent types of cancer, especially breast cancer, recommend the combination of these agents.^19^^,^^20^ Several studies have shown a large increase in the incidence of cardiovascular changes following cancer chemotherapy. Frequently, such changes are only clinically observed months or years after the use of these medications.^2^^,^^8^^,^^12^

The protocol for adjuvant and neoadjuvant treatment of breast cancer at the institution where the research was performed is based on the doxorubicin and cyclophosphamide regimen. The use of other regimens with taxane and 5-fluorouracil can be applied as an adjuvant and as a neoadjuvant; in our study, we chose not to include patients taking these drugs, because the incidence of HF is lower when compared to anthracyclics (5% to 35% of cases vs. 2% to 10%).^6^^,^^7^ Also, the number of patients using 5-fluorouracil-doxorubicin-cyclophosphamide, or 5-fluorouracil-epirubicin-cyclophosphamide in the institution is lower when compared to the AC regimen. As the incidence of cardiovascular toxicity with the use of trastuzumab alone is low in prospective clinical studies, ranging from 1% to 4%, commonly reversible if detected early, and with a good response to clinical treatment, we chose to exclude patients on their use.^21^^,^^22^

According to data from the World Health Organization (WHO), oncological diseases are currently the second largest cause of death in the world.^23^ The continuous therapeutic developments of the last decades allowed an increase in the survival of these patients. The adverse effects caused by chemotherapy, especially in the cardiovascular area, have become an important cause of morbidity and mortality in this population. It is estimated that the mortality rate among oncological patients who develop some cardiovascular event is high, with values higher than 60% when evaluated within 2 years. With this, cardiovascular disease has become a major cause of morbidity and mortality among cancer survivors.^24^^,^^25^

The main mechanism established for the increase in HF secondary to the use of doxorubicin is the direct myocardial damage of these agents (type I cardiotoxicity). The severity of the heart diseases triggered by chemotherapeutic agents seems to depend on the frequency and dose of the medicines administered; on the genetic characteristics; and on other cardiovascular comorbidities previously present.^11^^,^^26^ The mechanism related to myocardial damage seems to occur due to the production of free radicals from the reduction of the quinone group of B ring in the anthracyclic structure, leading to the production of superoxide anions and hydrogen peroxide, which saturate the antioxidant systems and react with the cellular structures, mainly in the membranes, causing cytotoxicity.^27^ However, recently, some authors have shown that, in addition to already established myocardial dysfunction, vascular alterations resulting from endothelial dysfunction also occur secondary to the use of anthracyclics and can be used as predictors for the cardiovascular toxicity induced by these agents.^5^^,^^28^ These changes may occur early, ^29^ and the mechanism proposed for these vascular changes is also related to the production of free radicals, with consequent cell death, or changes in the production of vasoactive endothelial factors.^2^^,^^8^^,^^30^

Some authors have already proposed the use of clinical tools to assess the vascular status of individuals undergoing antineoplastic therapies with anthracyclic drugs.^3^^,^^11^^,^^31^ These vascular changes could also justify an increase in the incidence of systemic arterial hypertension, atherosclerosis, and thromboembolic events in patients after chemotherapy.^11^^,^^32^^,^^33^ Early detection of dysfunctions in the vascular system is always difficult when non-histochemical methods are used, and it appears to develop from endothelial dysfunction, leading to progressive vascular remodeling.^28^ In addition, vascular changes could contribute to the increase of the preload and, consequently, to decrease of the cardiac output. Thus, in addition to direct myocardial damage, vascular alterations could, at least in part, be related to the decrease in LVEF in patients undergoing chemotherapy.

Several studies have tried to find early markers that can predict the occurrence of these changes in patients on chemotherapy with potential cardiovascular toxicity and, consequently, to detect patients at risk.

Currently, LVEF measurement by transthoracic Doppler echocardiogram is considered the main tool to monitor myocardial dysfunction induced by chemotherapy, and is used in several follow-up protocols.^7^^,^^34^^,^^35^ LVEF can also be measured by other techniques. Drafts et al.,^11^ in a study involving 53 patients who received anthracyclic chemotherapy, showed that changes in LVEF can be detected within 30 days of the beginning of chemotherapy sessions.^11^ However, these authors, in addition to using larger sampling, applied more accurate techniques, involving magnetic resonance imaging for the early detection of changes in ventricular volumes, compared to the classic Doppler echocardiogram routinely used in cancer treatment services and also in our study. Although other studies have shown a reduction in LVEF in patients at different times of treatment with these agents, our study was not able to show a significant reduction in LVEF between the values measured before the beginning of chemotherapy and in the post-4chemo. This fact may be due to the short period of patients follow-up, which does not allow the demonstration of a clinical change through this method - although structural and molecular microalterations have been shown early in this profile of patients, in the first months after treatment.^3^^,^^11^^,^^12^ There are studies suggesting that most cardiovascular alterations occur in the early stage, from the third month after the end of chemotherapy.^2^^,^^12^ Furthermore, because it is a pilot study, the reduced sampling may have contributed to this result.

The generic term “arterial stiffness” refers to changes in arterial mechanical properties, in response to acute or chronic phenomena, resulting in atherosclerosis and endothelial dysfunction, and correlating with increased cardiovascular morbidity and mortality.^9^ Currently, the best way to estimate AS is by measuring the PWV, obtained through the measurement of the time required for a wave formed by vascular distension to travel a certain distance between two points of an arterial segment.^9^ Thus, the greater the values of the PWV, the greater the AS. Some techniques do it with imaging tests, such as ultrasound techniques and magnetic resonance imaging with great precision. However, non-invasive devices, coupled with computerized systems, have been increasingly used for AS measurements.^12^

AS has been shown to be an early marker of cardiovascular diseases. A 2010 study showed, for the first time, a significant increase in aortic artery PWV, measured by magnetic resonance imaging, in patients after 4 months of chemotherapy with anthracyclics.^3^ In 2013, the same methodology was applied to patients in earlier stages of the same chemotherapy regimen, showing that it is possible to observe changes in PWV only 1 month after the administration of these agents.^11^ Despite the relevance of these studies in the predictability of AS changes in patients receiving anthracyclics, they apply methods that demand higher costs and specialized professionals for their technical performance. From 2010 onwards, portable devices appeared that were capable of simple estimation of AS of the brachial artery through oscillometric measurements of the upper limb, providing several hemodynamic data, which may be predictive markers of cardiovascular changes, such as PWV, augmentation index and cardiac index.^13^ Since this is an easy-to-use methodology, several studies have evaluated the potential of increased AS as a marker for cardiovascular diseases in various clinical conditions.^36^^,^^37^ Clinical studies have confirmed the validity of this instrument, which uses several algorithms to obtain hemodynamic variables such as PWV, which is the gold standard for assessing AS.^9^^,^^13^ With the same equipment, it is possible to measure central BP and other variables, which can be used to estimate arterial stiffness, but they are influenced by pathophysiological conditions, drugs and age, which make them less reliable.^38^^,^^39^

Due to the practicality of estimating AS through this method, our study proposed to evaluate the application of this methodology and correlate it with the data obtained by the LVEF through Doppler echocardiogram. The use of this tool could simplify the monitoring of cardiovascular toxicity induced by chemotherapy, since the use of Doppler echocardiography, as is routinely done for this purpose, is a method that requires higher cost, a qualified medical professional, and scheduled appointment at a specific time and place. This difficulty of access could reduce the guarantee of cardiotoxicity monitoring in patients who underwent chemotherapy.

In our study, all patients were monitored by this system at three different times (immediately before and after the first and fourth cycles of chemotherapy). In contrast to what was observed in other studies, which made these measurements early during chemotherapy, mainly by imaging tests,^3^^,^^11^ we were unable to show any significant statistical difference in the parameters evaluated at different times.

This study used the oscillometric method in the upper limb to show an increase in PWV and other hemodynamic parameters in 53 children with malignant tumors treated with anthracyclics.^29^ However, there was no difference in PWV after treatment with anthracyclines for a period of at least 1 year and without evaluation in the early stages of treatment. No other study in the literature that was researched evaluated any immediate changes in hemodynamic parameters shortly after the chemotherapy infusion of the AC regimen. Although we performed this assessment, we did not observe significant changes at this stage of treatment.

Our study showed an agreement between the parameters related to the estimation of AS by the oscillometric method and those observed in the LVEF values obtained from transthoracic Doppler echocardiography, in an early period of administration of chemotherapeutic agents. These data suggest that later studies, with longer follow-up and a larger sample, should test AS estimation through the method described as a practical and accessible tool for cardiovascular monitoring of breast cancer patients undergoing chemotherapy and using drugs with known cardiovascular toxicity potential.

## Conclusion

The application of measures of hemodynamic parameters that correlate with arterial stiffness, evaluated by oscillometric method of the upper limb, as well as the values of left ventricular ejection fraction, measured by transthoracic Doppler echocardiogram, was not changed in the early phase of chemotherapy - up to the fourth cycle of chemotherapy - in women with breast cancer on doxorubicin and cyclophosphamide.
